# Open Lichtenstein Hernioplasty Versus Laparoscopic Transabdominal Preperitoneal Mesh Repair: The Pain Factor

**DOI:** 10.7759/cureus.18282

**Published:** 2021-09-25

**Authors:** Chirag Pereira, Rakesh Rai

**Affiliations:** 1 General Surgery, Father Muller Medical College and Hospital, Mangalore, IND

**Keywords:** inguinal hernia, lichtenstein’s repair, post operative pain, laparoscopic tapp repair, inguinal hernia repair

## Abstract

Introduction

One of the most commonly performed surgery by a general surgeon is inguinal hernia repair. There have been numerous open surgical techniques and two laparoscopic techniques described in the literature for the treatment of inguinal hernias. The treatment outcome of all these surgeries remains the same which is reducing the hernia and preventing recurrence. Our aim was to compare laparoscopic versus open inguinal hernia repair with emphasis on postoperative pain.

Methods

One hundred and twenty patients with unilateral primary inguinal hernias were randomly divided into two groups. Each group included 60 patients. Group one was treated by open Lichtenstein repair, while the second group was treated by laparoscopic transabdominal preperitoneal (TAPP) mesh repair. The two groups were compared to assess the duration of surgery, postoperative pain, duration of hospital stay, return to normal activity, and work.

Results

Laparoscopic TAPP repair was found to have a longer operative time as compared to Liechtenstein open repair. In terms of other parameters such as postoperative pain duration of hospital stay, return to normal activity, and work the laparoscopic group was superior. After a one-year follow-up, none of the patients had any chronic pain or evidence of hernia recurrence.

Conclusion

Laparoscopic TAPP has a clear advantage over the conventional Liechtenstein open surgery especially in terms of reduced early post postoperative pain and return to normal activity.

## Introduction

Inguinal hernias are one of the most common surgeries performed by a general surgeon. There are various surgical techniques for the treatment of inguinal hernias that have been described in the literature as well as modifications that have been applied to these techniques. Lichtenstein tension-free hernioplasty was first described in 1989 and is considered as the gold standard treatment for inguinal hernias [1]. Apart from Liechtenstein open hernia repair, the laparoscopic approach has gained significant popularity in many centers throughout the world. The reason for its popularity is a shorter length of hospital stay and quicker resumption of normal activity [2,3].

Laparoscopic surgery, in general, is associated with less postoperative pain as it involves less surgical trauma in view of smaller incisions as compared to open surgery. Chronic pain is an important factor in the context of hernias as hernia repair involves the placement of a mesh during repair which can contribute to neuralgia. Hence, our study aims to compare laparoscopic transabdominal preperitoneal (TAPP) hernia repair with open Liechtenstein mesh repair with emphasis on early and late postoperative pain.

## Materials and methods

This prospective randomized study included 120 patients diagnosed with primary inguinal hernia. Patients were randomly divided into two groups. The first group of 60 patients underwent open Lichtenstein tension-free hernioplasty while the remaining patients in the second group underwent laparoscopic transabdominal preperitoneal (TAPP) mesh repair.

Inclusion criteria were patients with uncomplicated primary unilateral inguinal hernia aged from 18 to 90 years. Exclusion criteria included patients with bilateral inguinal hernias, complicated inguinal hernias, recurrent hernias, chronic liver or renal disease, unfit for major surgery, and history of chronic groin pain.

All the surgeries were performed by two surgeons experienced in laparoscopic and open repair in order to avoid bias. Patients were kept nil by mouth for about six hours prior to surgery and received a single dose of antibiotic prophylaxis half an hour before surgery. The type of anesthesia used was spinal anesthesia for open cases and general anesthesia for laparoscopic TAPP. Epidural anesthesia was not administered in any of the cases. In all patients, a monofilament polypropylene mesh was used and fixed appropriately.

During the postoperative period, all patients received intramuscular non-steroidal anti-inflammatory 12th hourly for one day postoperatively. The intramuscular injection was continued for an additional 24 hours if pain was severe [visual analogue scale (VAS) >IV]. Oral non-steroidal anti-inflammatory drugs (NSAIDs) were prescribed as and when required after 48 hours of surgery.

All the patients were encouraged for oral feeds after eight hours, initially, the feeds were sips of liquids followed by normal diet after the resolution of postoperative ileus (indicated by passing of flatus and normal bowel sounds on auscultation and return of appetite). The wounds were inspected for any seroma, hematoma or any infection. Patients were discharged after complete ambulation and tolerating normal diet.

The pain experienced by the patients in the postoperative period was graded according to the visual analogue scale (VAS) ranging from no pain to the worst possible pain on the scale of 0 to 10. It was recorded on the first and seventh postoperative day. After discharge, patients were encouraged to take normal diet and return to their normal activities as early as possible. After discharge, patients were followed up at one week, one month, three months, and six-month intervals. In the initial follow-up, the patients were evaluated for short-term complications like seroma or hematoma and wound infection. During subsequent visits, chronic pain at the operated site, return to normal activity were noted.

The two groups were compared regarding operative time, postoperative complications, postoperative pain, hospital stay, time to return to normal activity as well as work, and one-year recurrence rate. Data were analyzed using the chi-square test, a P value of <0.001 was considered significant. SPSS version 25 for mac was employed for statistical analysis.

## Results

The age of patients ranged from 21 to 84 years with the mean being 48.7 in the open group and 42.17 in the laparoscopic group. There were more males as compared to females in both the open group and laparoscopic group. The right inguinal hernia was more common as compared to the left (Table 1).

**Table 1:** Patient and hernia characteristics. TAPP: transabdominal preperitoneal.

	Lichtenstein open repair (n = 60)	Laparoscopic TAPP (n = 60)
Age (mean ± SD)	48.7 ± 7.22	42.17 ± 6.12
Sex		
Male	52 (86.7%)	56 (93.3%)
Female	8 (13.6%)	4 (6.7%)
Hernia site		
Right	38	39
Left	22	21

Laparoscopic TAPP had superior outcomes as compared to open Liechtenstein repair (Table 2). Laparoscopic TAPP has a significant longer operative time; 120.3 ± 3.1 minutes, compared to 64.7 ± 10.4 minutes for open mesh repair (P < 0.001). The mean pain score by the visual analogue scale after the first 24 hours was significantly less after laparoscopic TAPP compared to open mesh repair (2.73 ± 0.81 compared to 4.01 ± 0.64, P < 0.001). The duration of postoperative analgesia required in the laparoscopic group (1.57 ± 0.21 days) was significantly less than in the open group (2.93 ± 0.51); P < 0.001.

**Table 2:** Operative outcomes. TAPP: transabdominal preperitoneal.

	Lichtenstein open repair (n = 60)	Laparoscopic TAPP (n = 60)
Operative time (minutes) (mean ± SD)	64.7 ± 10.4	120.3 ± 3.1
Pain score after 24 hours (mean ± SD)	4.01 ± 0.64	2.73 ± 0.81
Pain score after seven days (mean ± SD)	2.98 ± 0.76	1.09 ± 0.1
Duration of hospital stay (days) (mean ± SD)	5.2 ± 0.41	3.07 ± 0.36
Return to normal activity (days) (mean ± SD)	13.20 ± 1.32	7.21 ± 1.12
Return to work (days) (mean ± SD)	18.55 ± 1.26	11.39 ± 1.09

A similar outcome was seen after one week where the mean pain score was 1.09 ± 0.12 in the laparoscopic TAPP group, compared to 2.98 ± 0.76 in the open group, P < 0.001. Only one patient in the open group had developed a seroma which was treated by aspiration. There were no other wound-related complications. Hospital stay was significantly shorter in the laparoscopic TAPP group compared to open repair (3.07 ± 0.36 days versus 5.2 ± 0.41 days, P < 0.001). Lap TAPP group had a significantly faster return to normal activity compared to the open group; (7.21 ± 1.12 days compared to 13.20 ± 1.32 days respectively, P < 0.001). Most of our patients that underwent surgery had an active work life and the laparoscopic TAPP group had a significantly shorter time for return to work compared to the open group (11.39 ± 1.09 versus 18.55 ± 1.26, P < 0.001). After a 12-week follow-up for chronic pain assessment, none of the patients had any complaints of postoperative pain or neuralgia.

## Discussion

The introduction of Lichtenstein tension-free hernioplasty considerably reduced the chances of hernia recurrence to as low as 1-4% making it a gold standard treatment. Since minimally invasive surgery is being used more commonly with most surgeries, it has the potential to be the new gold standard if done with expertise [4]. A total of 120 patients were involved in this study, of which 60 patients underwent laparoscopic TAPP and the remainder underwent Liechtenstein open hernioplasty. There was no statistically significant difference found in terms of age or sex in the study population.

In our study, the operative time for open surgery was significantly shorter as compared to laparoscopic TAPP. This may be due to the fact that setting up laparoscopic equipment takes a longer time as compared to open surgery. Scheuermann et al. [5] in their meta-analysis found that the operative time is longer in laparoscopic TAPP as compared to open surgery. Utiyama et al. [6] on the other hand found no significant difference. Studies have demonstrated that with specialization and experience, the operative time becomes clinically irrelevant [5,7].

In terms of postoperative pain and duration of hospital stay, laparoscopic TAPP was at a clear advantage. Since patients undergoing laparoscopic surgery had less postoperative pain, this ensured that they would mobilize early and it resulted in early discharge from the hospital as compared to the open group. One of the reasons for chronic pain following hernia surgeries is entrapment of the sensory nerves. Dividing or preserving these nerves during surgery has been debatable with no clear advantage of one over the other [8,9]. Laparoscopic TAPP involves a posterior approach, hence significantly reduces the chances of sensory nerve entrapment. In our study, none of the patients had any postoperative pain or neuralgia after the initial 12-week follow-up or after one-year of follow-up. Douek et al. [10], in their study, found that after a five-year follow-up 12 out of 242 patients that had undergone Liechtenstein open repair had paraesthesia and no such findings in the laparoscopic group.

Patients that underwent laparoscopic surgery in our study returned to normal activity and work quicker as compared to the open group which were similar to findings by Neumayer et al. [4]

In this study, we did not take the cost factor into consideration but since patients in the laparoscopic group were sent home early, this may bring down the overall cost of hospitalization but further studies need to be done to check its statistical significance.

One of the disadvantages of our study is that we did not have measures for long-term follow-up to look for recurrence as well as assess patients for pain after one-year following their surgery.

## Conclusions

Laparoscopic TAPP for primary unilateral inguinal hernia have a better outcome than open Lichtenstein repair, especially in terms of postoperative pain and early resumption of normal activity and work. 
